# Bicycle helmets are associated with fewer and less severe head injuries and fewer neurosurgical procedures

**DOI:** 10.1007/s00701-024-06294-6

**Published:** 2024-10-09

**Authors:** Ingar Næss, Mats Døving, Pål Galteland, Nils Oddvar Skaga, Torsten Eken, Eirik Helseth, Jon Ramm-Pettersen

**Affiliations:** 1 Department of Neurosurgery, Oslo University Hospital Ullevål, Nydalen, PO Box 4956, NO-0424 Oslo, Norway; 2 Department of Surgery/Orthopaedics, Finnmark Health Trust, Hammerfest, Norway; 3 Institute of Clinical Medicine, Faculty of Medicine, University of Oslo, Oslo, Norway; 4 Department of Maxillofacial Surgery, Oslo University Hospital Ullevål, Oslo, Norway; 5 Institute of Oral Biology, Faculty of Dentistry, University of Oslo, Oslo, Norway; 6 Department of Anaesthesiology and Intensive Care Medicine, Oslo University Hospital Ullevål, Oslo, Norway; 7 Department of Research and Development, Division of Emergencies and Critical Care, Oslo University Hospital, Oslo, Norway

**Keywords:** Bicycle, Helmet, Head injury, Blunt, Neurosurgical Procedure

## Abstract

**Purpose:**

This study explores the protective capabilities of bicycle helmets on serious head injury among bicyclists hospitalized in a Norwegian level 1 trauma centre.

**Method:**

Information on helmet use, demographic variables, Abbreviated Injury Scale (AIS) and surgical procedure codes was retrieved from the Oslo University Hospital Trauma Registry for patients with bicycle-related injuries from 2005 through 2016. Outcomes were serious head injury defined as maximum AIS severity score ≥ 3 in the AIS region Head, any cranial neurosurgical procedure, and 30-day mortality.

**Results:**

A total of 1256 hospitalized bicyclists were included. The median age was 41 years (quartiles 26–53), 73% were male, 5.3% had severe pre-injury comorbidities, and 54% wore a helmet at the time of injury. Serious head injury occurred in 30%, 9% underwent a cranial neurosurgical procedure, and 30-day mortality was 2%. Compared to non-helmeted bicyclists, helmeted bicyclists were older (43 years, quartiles 27–54, vs. 38 years, quartiles 23–53, *p* = 0.05), less often crashed during night-time (21% vs. 38%, *p* < 0.001), less frequently had serious head injury (22% vs. 38%, OR 0.29, 95% CI 0.22–0.39), and less often underwent cranial neurosurgery (6% vs. 14%, OR 0.36, 95% CI 0.24–0.54). No statistically significant difference in 30-day mortality between the two groups was found (1.5% vs. 2.9%, OR 0.50, 95% CI 0.22–1.11).

**Conclusion:**

Helmet use was associated with fewer and less severe head injuries and fewer neurosurgical procedures. This adds evidence to the protective capabilities of bicycle helmets.

## Introduction

Authorities run campaigns to increase the share of work commutes by bicycle and to make bicycling more attractive for the general population, and build bicycle-friendly facilities to increase perceived safety [24, 27, 37]. At the same time, a rise in the number of hospitalized bicyclists has been seen, and head injuries are prevalent [29].

Evidence suggests that wearing a helmet when bicycling reduces the risk of head injury [17, 33]. Still, the topic of helmet wearing is debated and many bicyclists omit wearing them [59]. Critics claim that helmeted bicyclists take more risks and that mandating helmets by law may discourage people from cycling [16, 42]. Other authors counter these claims, and there is evidence that mandating helmets by law reduces the number of head injuries [18, 26, 34]. Furthermore, any possible negative effects on the bicycling frequency from mandating helmets decrease over time [18, 34].

Helmet wearing for bicyclists in Norway is voluntary, but officials and road traffic safety organizations recommend using them [44]. Despite an observed increase in the rate of helmet wearing, head injury was the most frequent injury in bicyclists admitted to our trauma centre at Oslo University Hospital Ullevål (OUHU) [29], affecting six out of ten hospitalized bicyclists. Moreover, one tenth of all patients with traumatic brain injury hospitalized at OUHU are due to bicycle-related injuries [55].

Most previous studies on bicycle helmets have been performed in populations with either high or low rates of helmet wearing, had a low number of cases, or had non-optimal control groups [4, 8, 10, 58, 60, 61]. Therefore, we aimed to explore the potential effect of helmet use on preventing serious head injury, cranial neurosurgical procedures and 30-day mortality. We hypothesized less serious head injuries, less neurosurgical interventions and fewer deaths among helmeted than non-helmeted bicyclists.

## Methods

### Setting and population

OUHU is the level 1 trauma centre for the South-Eastern Norway Regional Health Authority, with a catchment area of 110 000 km^2^ containing 3.1 million inhabitants. Patients who sustain a potentially serious injury with an estimated transport time of less than 45 min, or those who are obviously in need of neurosurgical care independent of transport time, are brought directly to OUHU [30]. In addition, OUHU serves as a local acute care hospital for the citizens of Oslo. Patients who do not qualify for direct transport to OUHU receive initial treatment at other local acute care hospitals or outpatient clinics, before transfer to OUHU if necessary [43]. In the study period, there were 20 local hospitals in South-Eastern Norway with acute care function referring patients to OUHU. Thus, this is not a population-based study, but a selection of potentially seriously injured patients in Oslo or the region of South-Eastern Norway.

The Oslo University Hospital Trauma Registry (TR-OUH) prospectively includes all patients received by the trauma team, which is activated according to pre-defined criteria [30]. In addition, all patients with an injury severity score (ISS) ≥ 10, head injuries with an Abbreviated Injury Scale (AIS) severity code ≥ 3, and/or penetrating injuries to the head, neck, torso or proximal to the elbow or knee independent of ISS are included if admitted to OUHU within 24 h of injury [49].

### Bicycle cohort

The study was based on all patients admitted to OUHU due to bicycle-related injury from January 1, 2005, to December 31, 2016. Passengers on a bicycle at the time of crash were included, as well as patients declared dead on arrival at OUHU according to the Utstein template definition [40]. Bicyclists declared dead at the site of injury were not included. Pedestrians hit by a bicycle and bicyclists with no information regarding helmet use were excluded. Missing data was not imputed. The bicycle crashes were categorized into single bicycle crash, collision with a motorized vehicle, collision with another bicycle, collision with a pedestrian, or other.

### Variables extracted

The extracted variables contained information on demography, injury mechanism, injury severity, helmet use, treatment, and 30-day mortality verified with the Norwegian Population Registry. Pre-injury comorbidity was categorized according to the American Society of Anesthesiologists Physical Status Classification System (pre-injury ASA-PS) [6, 50]. Severe comorbidity was defined as pre-injury ASA-PS score ≥ 3. Night time was defined as 6:00 pm until 5:59 am. Information regarding alcohol influence was also extracted, although only tested on suspicion. Data on alcohol use was missing for 1045 (83%) patients and not analysed further.

Anatomical injury was coded according to the Abbreviated Injury Scale (AIS) 1990, Update 1998 [51]. Mild, moderate and serious head injury were defined as maximum AIS (mAIS) 1, 2, and ≥ 3, respectively, in the AIS region Head. Overall injury severity was assessed with Injury Severity Score (ISS), which is calculated from the most severe AIS injury code from three different body regions, and with the New Injury Severity Score (NISS), which is calculated from the three most severe AIS injury codes irrespective of body regions [7, 35].

To study the frequency of cranial neurosurgery, the Nordic Medico-Statistical Committee (NOMESCO) classification of surgical procedures (NCSP) was utilized [31]. Codes with the prefix AA were extracted. Individual NCSP codes were only counted once per patient irrespective of the actual number of procedures performed.

### Statistics

Data analysis was undertaken using IBM SPSS statistics version 28 (IBM Corp., Armonk, NY). Descriptive statistics are presented with absolute number and percentage, or median and quartiles. To detect group differences, Pearson's χ^2^ test was applied for categorical variables, or Fisher's Exact Test if the sample size was < 5, and the Mann–Whitney U test for continuous variables due to generally skewed distributions. Effects of helmet use vs. non-use on a set of response variables were evaluated in logistic regression analyses. Response variables were head injury vs. no head injury in the total population and stratified by head injury severity, any neurosurgical intervention vs. no neurosurgical intervention, and 30-day death vs. survival. Effects of helmet use on serious head injury were further studied stratified by age group and by type of crash. Odds ratios are reported with 95% confidence intervals (CI). Odds ratios adjusted for sex, pre-injury ASA-PS score, and age if appropriate (aOR) are also reported.

## Results

A total of 1543 bicyclists were admitted to OUHU during the 12-year study period; the 1256 bicyclists with information regarding helmet use constituted the study population. The median age was 41 years, 73% were male, and 5.3% had severe pre-injury comorbidity. Single bicycle crashes occurred in 67%, 27% crashed during night-time, and 54% wore a helmet at the time of injury (Table 1).

**Table 1:** Basic characteristics of helmeted and non-helmeted bicyclists

	Total *N* = 1256	Helmet *N* = 678	No helmet *N* = 578	*p*-value
Age				
	41 (26–53)	43 (27–54)	38 (23–53)	**0.05**
Sex				
Male	918 (73)	499 (74)	419 (72)	0.66
Female	338 (27)	179 (26)	159 (28)	
Comorbidity				
Pre-injury ASA-PS 1–2	1189 (95)	651 (96)	538 (93)	**0.02**
Pre-injury ASA-PS 3–4	67 (5.3)	27 (4.0)	40 (6.9)	
Time of crash^1^				
Day-time (06–18)	857 (71)	517 (79)	340 (62)	** < 0.001**
Night-time (18–06)	343 (29)	138 (21)	205 (38)	
Type of crash				
Single bicycle crash	847 (67)	456 (67)	391 (68)	0.24
Collision with motorized vehicle	340 (27)	178 (26)	162 (28)	
Collision with another bicycle	57 (5)	38 (6)	19 (3)	
Collision with pedestrian	6 (0.5)	2 (0.3)	4 (0.7)	
Other	6 (0.5)	4 (0.6)	2 (0.3)	
Consciousness				
GCS 15	931 (74)	548 (81)	383 (66)	** < 0.001**
GCS 13–14	185 (14)	76 (11)	109 (19)	
GCS 9–12	47 (3.7)	16 (2.4)	31 (5.4)	
GCS 3–8	93 (7.4)	38 (5.6)	55 (10)	
Injury severity				
ISS	10 (5–17)	10 (5–17)	10 (5–17)	0.50
NISS	12 (5–22)	12 (5–22)	12 (5–27)	0.09

Numbers are count with percent or median with quartiles

^1^Time missing for 23 helmeted and 33 non-helmeted cyclists

*GCS* Glasgow Coma Scale

*ISS* Injury Severity Score

*NISS* New Injury Severity Score

*ASA-PS* American Association of Anasthesiologists Physical Status Classification System

GCS ≤ 14 was registered in 26% of the bicyclists (Table 1). The aggregate number of AIS codes was 4906, with a median of 3 (quartiles 2–5, range 1–26) codes per bicyclist. Two or more AIS codes were present in 1156 (88%) patients, while 536 (43%) patients had injuries in two or more ISS body regions (excluding external injuries). The injury rates for each ISS body region were: 892 (71%) had head and neck injuries, 348 (28%) had extremity or pelvic girdle injuries, 336 (27%) had facial injuries, 313 (25%) had chest injuries, 96 (8%) had injuries to the abdomen or pelvic content, and 1154 (92%) had external injuries. ISS ≥ 9 was present in 772 (61%) bicyclists; 407 (32%) had ISS ≥ 16. Cranial neurosurgery was performed in 116 (9%) injured bicyclists (Table 2).

**Table 2:** The association of helmet wearing and head injury, cranial neurosurgical procedures and mortality, adjusted for age, sex and pre-injury ASA-PS score

	Total *N* = 1256	Helmet *N* = 678	No helmet *N* = 578	OR (95% CI)	*p*-value	aOR (95% CI)	*p*-value
Head injury							
Mild and moderate	453 (36)	230 (34)	223 (39)	0.46 (0.35–0.61)	** < 0.001**	0.47 (0.35–0.61)	** < 0.001**
Serious	372 (30)	150 (22)	222 (38)	0.30 (0.23–0.40)	** < 0.001**	0.29 (0.22–0.39)	** < 0.001**
Any head injury	825 (66)	380 (56)	445 (77)	0.38 (0.30–0.49)	** < 0.001**	0.38 (0.30–0.49)	** < 0.001**
Any cranial neurosurgery	116 (9.2)	37 (3.2)	79 (14)	0.37 (0.24–0.55)	** < 0.001**	0.36 (0.24–0.54)	** < 0.001**
Mortality	27 (2.1)	10 (1.5)	17 (2.9)	0.49 (0.22–1.09)	0.08	0.50 (0.22–1.11)	0.09

Multivariable logistic regression. No helmet is the reference, and bicyclists with head injury were compared to bicyclists with no head injury. Group sizes are shown as count with percent. *OR* odds ratio; *aOR* odds ratio adjusted for age, sex and pre-injury ASA-PS score; *CI* confidence interval

**Fig. 1:**
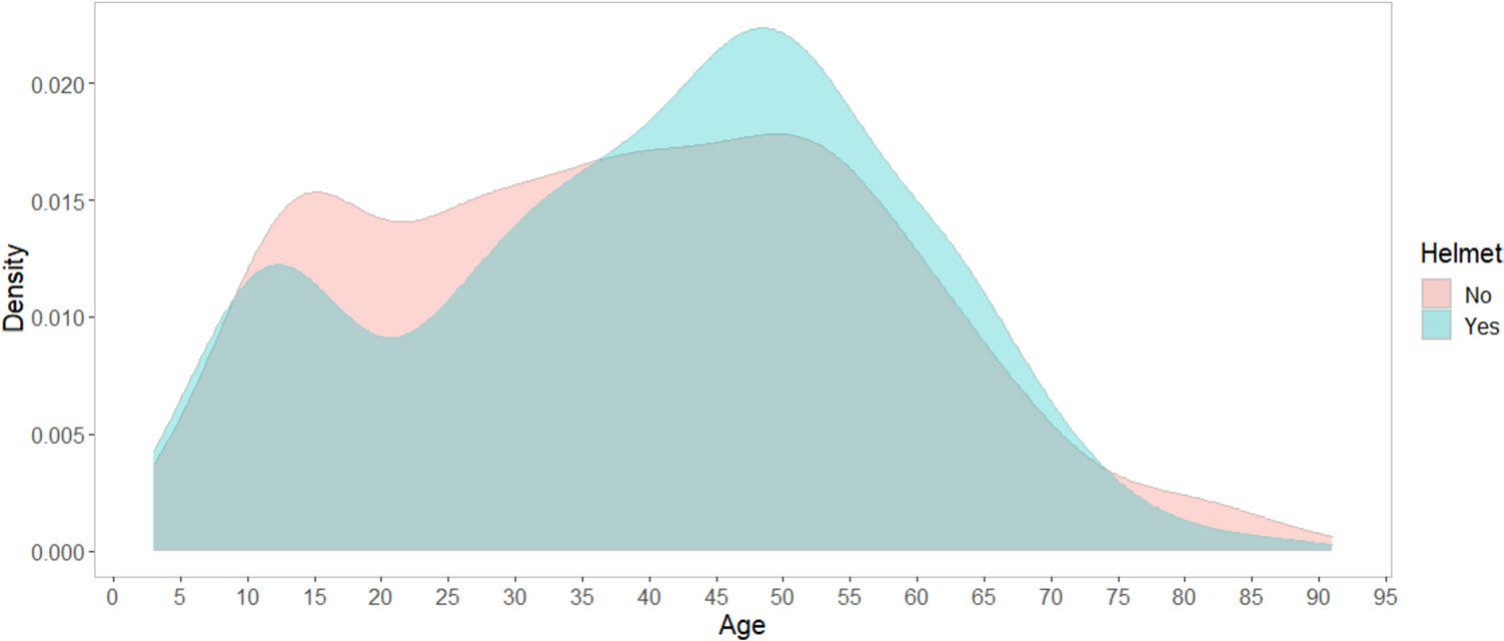
The distribution of helmet use and age (*N* = 1256). Helmeted bicyclists in blue and non-helmeted bicyclists in red

Helmeted bicyclists were older, less likely to have severe pre-injury comorbidity, less likely to crash during night-time, and less often had reduced GCS than non-helmeted bicyclists (Table 1, Fig. 1). No statistically significant difference was found between helmeted and non-helmeted bicyclists with respect to sex, type of crash, ISS, and NISS (Table 1).

A statistically significant 62% odds reduction for any head injury was found for helmeted compared to non-helmeted bicyclists (Table 2). Helmeted bicyclists had a larger reduction in OR for sustaining a serious head injury compared to sustaining a mild or moderate head injury (aOR 0.63, 95% CI 0.48–0.84, *p* = 0.001, Table 2). A significantly reduced OR for sustaining a serious head injury was seen in all age groups, except for those aged 70 and older (Table 3).

**Table 3:** The association of helmet wearing and serious head injury (*N* = 372) stratified by age groups, adjusted for sex and pre-injury ASA-PS score

Age	Total *N* = 372	Helmet *N* = 150	No helmet *N* = 222	OR (95% CI)	*p*-value	aOR (95% CI)	*p*-value
0–9	10 (2.7)	2 (1.3)	8 (3.6)	0.13 (0.02–0.75)	**0.02**	0.12 (0.02–0.79)	**0.02**
10–19	54 (15)	20 (13)	34 (15)	0.31 (0.14–0.70)	**0.005**	0.31 (0.14–0.71)	**0.005**
20–29	38 (10)	11 (7.3)	27 (12)	0.28 (0.12–0.66)	**0.004**	0.26 (0.11–0.61)	**0.002**
30–39	44 (12)	20 (13)	24 (11)	0.36 (0.17–0.78)	**0.01**	0.37 (0.17–0.80)	**0.01**
40–49	67 (18)	32 (21)	35 (16)	0.30 (0.15–0.59)	** < 0.001**	0.28 (0.14–0.56)	** < 0.001**
50–59	76 (20)	31 (21)	45 (20)	0.31 (0.16–0.60)	** < 0.001**	0.30 (0.15–0.58)	** < 0.001**
60–69	50 (13)	22 (15)	28 (13)	0.23 (0.09–0.57)	**0.001**	0.24 (0.10–0.58)	**0.002**
70 +	33 (8.9)	12 (8.0)	21 (9.5)	0.25 (0.05–1.13)	0.07	0.19 (0.03–1.12)	0.07

Multivariable logistic regression. No helmet is the reference, and bicyclists with serious head injury were compared to bicyclists with no head injury. Group sizes are shown as count with percent. *OR* odds ratio; *aOR* odds ratio adjusted for sex and pre-injury ASA-PS score; *CI* confidence interval

Helmet use was associated with a significant odds reduction for serious head injury irrespective of type of bicycle crash. The highest reduction was seen for bicycle vs. bicycle collision, followed by single bicycle crash and then collision with a motorized vehicle (Table 4).

**Table 4:** The association of helmet wearing and serious head injury (*N* = 372) stratified by type of crash, adjusted for age, sex and pre-injury ASA-PS score

Type of crash	Total *N* = 372	Helmet *N* = 150	No helmet *N* = 222	OR (95% CI)	*p*-value	aOR (95% CI)	*p*-value
Single bicycle crash	259 (70)	104 (69)	155 (70)	0.28 (0.20–0.40)	** < 0.001**	0.28 (0.20–0.40)	** < 0.001**
Collision with motorized vehicle	85 (23)	35 (23)	50 (23)	0.40 (0.23–0.71)	**0.002**	0.37 (0.20–0.66)	** < 0.001**
Collision with another bicycle	24 (6.5)	10 (6.7)	14 (6.3)	0.13 (0.03–0.55)	**0.006**	0.06 (0.01–0.39)	**0.003**
Collision with pedestrian	3 (0.8)	0 (0)	3 (1.4)	-		-	
Other	1 (0.3)	1 (0.7)	0 (0)	-		-	

Multivariable logistic regression. No helmet is the reference, and bicyclists with serious head injury were compared to bicyclists with no head injury. Group sizes are shown as count with percent. *OR* odds ratio; *aOR* odds ratio adjusted for age, sex and pre-injury ASA-PS score; *CI* confidence interval

Helmeted bicyclists had an adjusted odds reduction of 64% for having any cranial neurosurgical procedure compared to non-helmeted bicyclists (Table 2). The most common procedure was insertion of an intracranial pressure monitoring device, followed by external drainage of cerebrospinal fluid and evacuation of acute subdural haematoma (Table 5).

**Table 5:** Patients subjected to neurosurgical procedures (*N* = 116)

NOMESCO classification of surgical procedures code*:	Total *N* = 116	Helmet *N* = 37	No helmet *N* = 79	χ^2^/Fisher	aOR (95% CI)	*p*-value
Insertion of ICP monitoring device	78 (67)	29 (78)	49 (62)	**0.002**	0.48 (0.30–0.78)	**0.003**
Evacuation of epidural haematoma	17 (15)	2 (5.4)	15 (19)	** < 0.001**	0.11 (0.03–0.50)	**0.004**
Evacuation of acute subdural haematoma	21 (18)	5 (14)	16 (20)	**0.005**	0.28 (0.10–0.77)	**0.01**
Evacuation of chronic subdural haematoma	7 (6.0)	1 (3)	6 (7.6)	0.053	0.14 (0.02–1.2)	0.07
Evacuation of traumatic intracerebral haematoma	14 (12)	1 (3)	13 (17)	** < 0.001**	0.06 (0.01–0.50)	**0.008**
Revision of fracture of skull	11 (9.5)	0 (0)	11 (14)	** < 0.001**	-	0.99
External drainage of ventricle of brain	26 (22)	9 (24)	17 (22)	**0.045**	0.45 (0.20–1.02)	0.054
Cranioplasty	14 (12)	3 (8.1)	11 (14)	**0.02**	0.24 (0.07–0.85)	**0.03**
Repair of dura	18 (16)	2 (5.4)	16 (20)	** < 0.001**	0.11 (0.03–0.49)	**0.004**
Other AA codes	15	8	7	0.96		
All AA codes	221	60	161	** < 0.001**		

Multivariable logistic regression. No helmet is the reference, and bicyclists with head injury were compared to bicyclists with no head injury. Group sizes are shown as count with percent. *ICP* intracranial pressure; *aOR* odds ratio adjusted for age, sex and pre-injury ASA-PS score; *CI* confidence interval

^*^Any AA code was only applied to a patient once, although a bicyclist may have undergone the procedure several times

A total of 27 (2.1%) bicyclists died within 30 days of injury (Table 2); 30-day mortality for helmeted bicyclist was 1.5% and for non-helmeted bicyclists 2.9% (*p* = 0.09).

## Discussion

This study compared head injuries, cranial neurosurgical procedures, and mortality among helmeted and non-helmeted bicyclists admitted to a Norwegian level 1 trauma centre. Helmeted bicyclists had lower rates of head injuries and cranial neurosurgical procedures compared to non-helmeted bicyclists. No statistically significant difference in mortality was found between the two groups.

The median age of the injured bicyclists in our study was 41 years and 5.3% had severe pre-injury comorbidities. Thus, the typical injured bicyclist was young and with little or no comorbidities. This is different from the typical western world patient hospitalized with a traumatic brain injury (TBI) or cervical spine fracture, which are older and have more comorbidities [55, 57]. In general, young patients have better physiologically reserve capacity than older patients, and they will likely recover faster and better than older patients. Advanced acute health care and rehabilitation are expensive, and in most countries the resources are limited. It is understandable that young patients sometimes will have priority before old patients with severe comorbidities and shorter life-expectancy [54]. Bicycle-related traumatic brain injury represents a burden to the society and can be detrimental to the life of the patient, both physically and emotionally [45, 62]. Thus, bicycle-related traumatic brain injury is important to prevent.

In the present study, 54% of the bicyclists wore helmets, which is similar to what was observed among injured bicyclists in an outpatient clinic in Oslo towards the end of the study period [28]. The frequency of helmet wearing varies among countries, as documented in studies on hospitalized bicyclists in the Netherlands (4.4–7.7%), France (14%), the United States (22–25.1%), Sweden (43.5%), Denmark (36.4–55.4%), the United Kingdom (61.5%) and Australia (72.3%) [4, 10, 20, 46, 47, 60, 61]. Of these, Australia is the only country where bicycling helmet use is mandated and wearing rates at 98% have been seen [9]. Although not mandatory in Norway, helmet wearing among hospitalized bicyclists increased between 2005 and 2016 [29].

Some argue that helmet-wearing bicyclists engage in more risk-taking behaviour due to increased perceived safety [15, 16]. So far, evidence in favour of this risk compensation theory is sparse [13]. In fact, it has been shown that helmeted bicyclists more often wear other safety equipment and that they are more cautious in traffic compared to non-helmeted bicyclists [19].

Mandating helmets by law increases wearing rates of bicycle helmets and most likely reduces the number of head injuries [22, 23, 26, 34]. In the Netherlands, a reduction of 46 deaths and 2942 cases of traumatic brain injury per year has been estimated if a law enforcing helmet use were introduced [39]. Furthermore, any negative effects of mandating helmet use are most uncertain [18, 26, 34]. The present study found less serious head injuries for helmeted compared to non-helmeted bicyclists. Therefore, to prevent serious head injuries, increasing the rate of helmet wearing is desirable and mandating helmets by law could be considered among other safety measures [38].

Helmet use was associated with higher age, less severe pre-injury comorbidity, and daytime bicycling. Higher age among helmet users has also been found by other authors [4, 10]. Previous studies show that bicycling at night-time is associated with low rates of helmet wearing, higher incidence of alcohol influence, and an increased risk of head injury [3, 5, 21]. Seemingly, young bicyclists and those bicycling at night-time tend to avoid helmet-wearing and could be target groups for campaigns for helmet wearing.

We found serious head injury for 22% of helmeted bicyclists compared to 38% of those without helmets. In a retrospective study from the NHS England Trauma Audit and Research Network dataset, the corresponding numbers were 19% and 48% [10]. Baschera et al. reported lower rate of head injuries among injured bicyclists in their Australian trauma centre than in comparable studies from populations with lower rates of helmet wearing and without helmet legislation [8]. In our study, the odds reduction for serious head injury for helmet users varied across different injury mechanisms, namely 72% for single bicycle crash, 63% for collision with a motorized vehicle, and 94% for collision with another bicycle. In line with our results, a meta-analysis also reported larger effect size for helmets in single bicycle crashes compared to bicycle-collisions with motorized vehicles [17]. Thus, helmets appear to protect from head injuries independent of crash mechanism [4].

Cranial neurosurgical procedures can be used as markers for head injury severity [56]. We found that helmeted bicyclists had significantly reduced odds for cranial neurosurgical procedures [10, 14]. In addition, a study on hospitalized bicyclists with intracranial haemorrhage found a lower frequency of craniotomies for helmeted compared to non-helmeted bicyclists [20]. Most bicycle-related deaths occur due to head injuries [36]. In our study, there was no statistically significant difference in 30-day mortality between the helmeted and non-helmeted groups. This is in contrast to other authors who have found helmet use to be associated with lower mortality [10, 46]. In a comparison of Dutch and Australian bicyclists admitted to trauma centres, the Dutch bicyclists had more cases of serious head injury and a higher mortality rate. The authors noted low rates of helmet use in the Dutch population, but the patients were also older [60]. Increased age and pre-injury comorbidity are associated with higher mortality rates in trauma patients, and this pertains to bicycling as well [12, 50]. Even so, according to our results and previously suggested, helmets seem beneficial for all age groups [52, 53].

Comparable studies vary in their choice of control group. The present study used bicyclists without head injury as controls, other authors have included mild and moderate head injury, and some have used controls with no injuries to the head, neck or face [4, 10]. In a meta-analysis on helmet use in bicycling accidents, the authors state that the latter method is preferable. Yet, the results in that meta-analysis were unchanged in a reanalysis including only studies with the preferred control group [33]. Furthermore, the results across two meta-analyses on helmet use are consistent, reporting odds reductions for serious head injury for helmeted compared to non-helmeted bicyclists of 60% and 69%, respectively [17, 33]. Of note, the term “serious head injury” in the meta-analyses was put together from varied definitions among the included studies, such as AIS ≥ 3, traumatic brain injury, skull fractures, loss of consciousness, or head injuries reported as serious or severe in the original study. Even so, the results of these meta-analyses are in line with our finding of a 71% odds reduction for serious head injury among helmeted bicyclists.

Strengths of the present study are the large, prospectively collected 12-year material and the relatively equal distribution of bicyclists with and without helmets. In contrast to comparable studies, there was no statistically significant difference in overall injury severity measured as NISS between helmet and non-helmeted bicyclists; we believe this enhances comparability between the two groups [10]. Another strength is the precise anatomical definition of injury severity. Although subject to inter-rater variability, the AIS is a widely recognized system and ensures transparency and reproducibility [41].

The study has limitations. Nearly 20% of the cases in the original cohort were excluded due to unknown helmet status, although fewer than in comparable studies [10, 29]. Another limitation is that alcohol influence could not be assessed due to low test rates. Alcohol is a potential confounder and is associated with both head injury and non-helmet wearing [3, 48]. Still, there is evidence that alcohol intoxication does not oppose the protective effect from helmet use [32]. Further, there were only 10 patients in the age group 70 + with no head injury, which may explain the non-significant association in the logistic regression analysis of helmet use and mortality for the oldest bicyclists. Experimental studies indicate that new helmet designs incorporating anti-rotational technology, including airbag helmets, offer superior protection against traumatic brain injury by reducing rotational forces during oblique impacts compared to conventional helmets. However, clinical studies validating these findings are still lacking [1, 2, 25]. Notably, the company behind the airbag helmet Hövding filed for bankruptcy after sales were temporarily suspended by the Swedish Consumer Agency due to incomplete testing and safety concerns [11]. Unfortunately, assessment of the quality of different bicycle helmet designs was beyond the scope of this study.

Selection bias exists as OUHU is the only centre in the region offering neurosurgical trauma care. Therefore, it is likely that most bicyclists who sustained serious head injuries within the region were transferred to OUHU. Hence, an overrepresentation of head injuries and consequently of non-helmeted bicyclists might be present. Moreover, the study did not include data from bicyclists who were involved in a crash but avoided injury because of helmet use. Thus, the beneficial association between helmet wearing and head injury might be underestimated [4, 10, 17].

## Conclusions

For bicyclists hospitalized to a Norwegian level 1 trauma centre, helmet wearing was associated with a lower rate of head injuries for all head injury severities compared to not wearing helmets. The associated reduction in head injuries was greater for serious than mild and moderate head injuries. Those wearing helmets also underwent fewer cranial neurosurgical procedures than non-helmeted bicyclists. In conclusion, wearing helmets protects bicyclists from head injury. Therefore, authorities should consider means to increase helmet use.

## Data Availability

The de-identified data that support the findings of this study are available from the corresponding author upon reasonable request and with permission of the Oslo University Hospital Data Protection Officer.
